# Biochemical Characterisation of Sis: A Distinct Thermophilic PETase with Enhanced NanoPET Substrate Hydrolysis and Thermal Stability

**DOI:** 10.3390/ijms25158120

**Published:** 2024-07-25

**Authors:** Carmen Ercolano, Roberta Iacono, Valeria Cafaro, Elio Pizzo, Donato Giovannelli, Golo Feuerriegel, Wolfgang R. Streit, Andrea Strazzulli, Marco Moracci

**Affiliations:** 1 Department of Biology, University of Naples Federico II, Complesso Universitario di Monte S. Angelo, Via Cinthia 21, 80126 Naples, Italy; carmen.ercolano@unina.it (C.E.); roberta.iacono@unina.it (R.I.); valeria.cafaro@unina.it (V.C.); elipizzo@unina.it (E.P.); donato.giovannelli@unina.it (D.G.); marco.moracci@unina.it (M.M.); 2 National Biodiversity Future Center (NBFC), 90133 Palermo, Italy; 3 Centro Servizi Metrologici e Tecnologici Avanzati (CeSMA), University of Naples Federico II, 80126 Naples, Italy; 4 Institute for Marine Biological Resources and Biotechnologies, Italian National Research Council, CNR-IRBIM, 60125 Ancona, Italy; 5 Department of Marine and Coastal Science, Rutgers University, New Brunswick, NJ 08901, USA; 6 Marine Chemistry and Geochemistry Department, Woods Hole Oceanographic Institution, Woods Hole, MA 02543, USA; 7 Earth-Life Science Institute (ELSI), Tokyo Institute of Technology, Tokyo 152-8552, Japan; 8 Department of Microbiology and Biotechnology, University of Hamburg, 22609 Hamburg, Germany; golo.feuerriegel@uni-hamburg.de (G.F.); wolfgang.streit@uni-hamburg.de (W.R.S.)

**Keywords:** PET hydrolysis, enzyme discovery, thermozymes

## Abstract

Polyethylene terephthalate (PET) degradation by enzymatic hydrolysis is significant for addressing plastic pollution and fostering sustainable waste management practices. Identifying thermophilic and thermostable PET hydrolases is particularly crucial for industrial bioprocesses, where elevated temperatures may enhance enzymatic efficiency and process kinetics. In this study, we present the discovery of a novel thermophilic and thermostable PETase enzyme named Sis, obtained through metagenomic sequence-based analysis. Sis exhibits robust activity on nanoPET substrates, demonstrating effectiveness at temperatures up to 70 °C and displaying exceptional thermal stability with a melting temperature (T_m_) of 82 °C. Phylogenetically distinct from previously characterised PET hydrolases, Sis represents a valuable addition to the repertoire of enzymes suitable for PET degradation.

## 1. Introduction

Polyethylene terephthalate (PET) is a widely used plastic material that poses serious environmental problems due to its persistence and accumulation in natural ecosystems. Enzymatic degradation of PET by specific hydrolases (PETases, EC 3.1.1.101) is a promising strategy for PET recycling and upcycling [1]. However, enzymatic hydrolysis is influenced by several factors, such as the substrate crystallinity, the reaction temperature, and the enzyme properties. Therefore, the discovery of new thermophilic PETases with enhanced performance and stability is of great interest for the development of efficient and sustainable biocatalysts for PET hydrolysis [2].

PET hydrolases are a set of enzymes that belong to the α/β hydrolase family. Despite their low sequence identity, they exhibit remarkably similar folds. Various types of hydrolases capable of degrading PET have been identified as cutinases (EC 3.1.1.74), lipases (EC 3.1.1.3), carboxylesterases (EC 3.1.1.1), PETases (EC 3.1.1.101), MHETases (EC 3.1.1.102), and esterases (EC 3.1.1.1) [3]. Among them, PETases catalyse the hydrolysis of the ester bonds of PET, producing terephthalic acid (TPA) and ethylene glycol (EG) as monomers and bis-hydroxyethyl terephthalate (BHET) and mono-2-hydroxyethyl terephthalate (MHET) as oligomers [4].

PET hydrolases are classified based on their sequence into two types: type I and type II. Type I enzymes possess a single C-terminal disulfide bond and the extended loop found in *Is*PETase [5,6]. In contrast, type II enzymes possess an additional disulfide bond adjacent to the active site, connecting specific loops containing catalytic residues [7,8]. In addition, type II enzymes are divided into two subtypes, namely types IIa and IIb [9,10]. This classification was proposed by comparing their features with those of PETase from *Ideonella sakaiensis* [11], *Is*PETase here, which belongs to type IIb PET hydrolases. Regardless of their types, these hydrolases share common features, including a catalytic triad (Ser-His-Asp), an aromatic amino acid (Phe or Tyr) located between the β3-strand and the α1-helix, and a Met residue in the α4-helix, close by the catalytic Ser, forming the oxyanion hole crucial for transition state stabilisation. In addition, PET hydrolases present a GXSXG motif that plays a critical role in the enzyme’s function, which includes the catalytic Ser residue. In type I PETases, such as the Leaf-branch Compost Cutinase (LCC) enzyme [5], the first X is a His residue, while, in other types, it is a Trp. Additionally, the second X is a Met, which is strictly conserved across all bacterial PET hydrolase sequences. Moreover, the conservation of the C-terminal disulfide bond is crucial for maintaining the overall stability of PET hydrolases. This bond, crucial for the functionality and structural integrity of the enzyme, connects the terminal α6-helix with the β9-strand [12]. For an in-depth analysis of the structure–function dynamics of PET hydrolases, delve into the high-quality review by Tournier et al. in 2023 [2].

The discovery and screening of novel PET hydrolases rely on different techniques, such as genomic and metagenomic approaches. Genomic methods involve the isolation and cultivation of microorganisms and genome sequencing and annotation to identify novel PETases [13]. Differently, metagenomic approaches involve the extraction and analysis of DNA from environmental samples, such as soil, compost, or marine sediments. By using functional or sequence-based approaches, genes encoding for potential PET hydrolases can be identified and expressed in suitable hosts or using in vitro cell-free expression systems for further characterisation [5,14,15]. All the characterised PET hydrolases are then reported in the PAZy (Plastics-Active enZymes) database (https://pazy.eu/doku.php), a comprehensive and manually curated resource that lists only biochemically or structurally characterised enzymes known to degrade plastics, providing information on the global annual production of various polymers and on the number of enzymes known to act on them [16,17].

One of the challenges for the enzymatic hydrolysis of PET is represented by the high crystallinity of the substrate, which limits the accessibility and activity of the enzymes. Indeed, PET has a glass transition temperature T_g_ of −75 °C above which the polymer chains become more flexible and susceptible to hydrolysis [18]. Therefore, thermophilic and thermostable PET hydrolases, which are stable and active at high temperatures, are desirable for efficient PET degradation. So far, all thermostable PET hydrolases (melting temperature T_m_ > 55 °C) are classified as type I, with the exception of enzyme PET2, which belongs to type IIa, a subgroup of mesophilic PET hydrolases (T_m_ < 55 °C) classified as type II [7,15] (Table 1).

Here, we report the discovery and characterisation of a novel thermophilic and thermostable PETase named Sis, identified through in silico methods in an environmental metagenomic sample. Sis, while structurally resembling typical PET hydrolases, is phylogenetically distinct from previously reported types of such class of enzymes. Nevertheless, it exhibits efficient activity on BHET and PET nanoparticles (nanoPET) up to 70 °C, with a T_m_ of 82 °C.

## 2. Results and Discussion

### 2.1. Identification and Sequence Analysis of Sis

**Figure 1:**
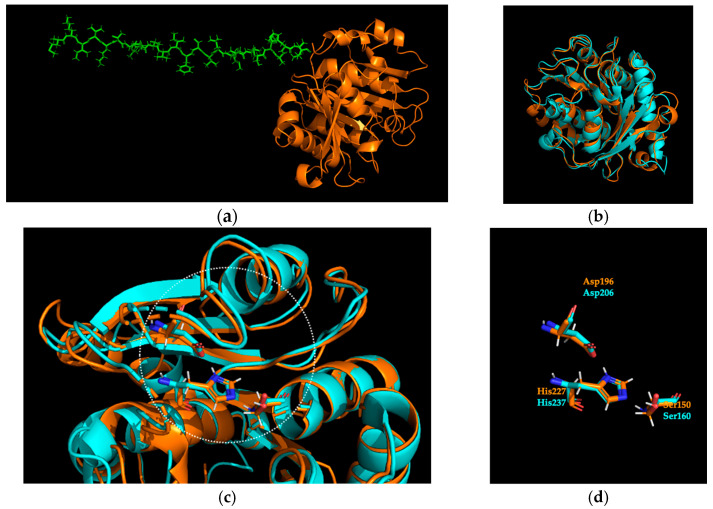
Structural analysis of Sis. (**a**) Sis model (orange), including the signal peptide (green). (**b**) Structural superimposition of Sis (orange) and *Is*PETase (PDB 6QGC, cyan). (**c**,**d**) Close-up of the active site, with the superposition of catalytic residues of Sis (orange) and *Is*PETase (cyan). Non-carbon atoms are coloured according to the CPK colouring convention.

In order to identify novel PET hydrolysing activities, we performed a search against private unpublished metagenomics datasets exploiting a Hidden Markov Model (HMM) created starting from the PET hydrolysing enzymes (PHEs) sequences available in PAZy online database, as previously described [7,16,19]. Based on the percentages of identity and the 3D structure prediction by using ColabFold v1.5.5 [20] (Figure 1a), we selected Sis from a metagenomic sample obtained by a geothermal site (36 °C, pH 9.8), having an HMM score of 279.3. Sis showed 52% and 91% identity vs. PAZy and *NR* databases (in February 2024), respectively (Figures S1 and S2). In particular, the thermophilic Cutinase 1 from *Thermobifida cellulosilytica* (Thc_Cut1, ADV92526.1) resulted as the closer PAZy reference [21,22]. Differently, the analysis of the *NR* database has shown the closest references in a cluster of three hypothetical proteins belonging to the order Syntrophales within the phylum Thermodesulfobacteriota.

The detailed sequence analysis by pairwise alignment with *Is*PETase (Figures S3 and S4), selected as the structural reference [11], confirmed the presence of the residues forming the catalytic triad, the oxyanion hole, the aromatic clamp, the disulfide bond, and the consensus motif GXSXG, which represents the common trait of α/β serine hydrolases already observed in the other characterised cutinase-like PETases (Figure S4) [23].

**Figure 2:**
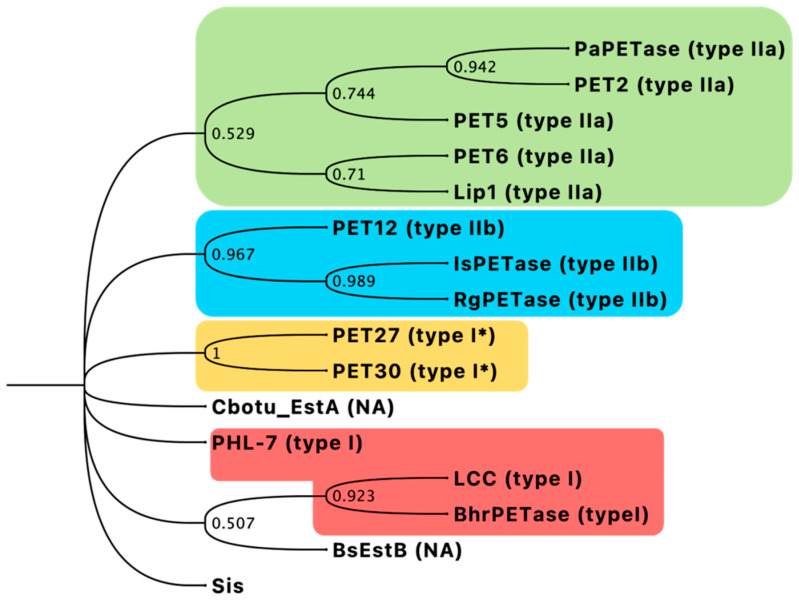
Phylogenetic tree of Sis with representatives of bacterial PET hydrolases of types I, I*, IIa, and IIb in red, orange, green, and blue, respectively. NA: not assigned. Bootstrap values are indicated at respective nodes.

In addition, the phylogenetic analysis of representative PET hydrolase sequences from different types showed that Sis is distinct from PHEs classified as type I/I* and those belonging to type IIa/IIb. Moreover, Sis also does not cluster with the *p*-nitrobenzylesterase from *Bacillus subtilis* BsEstB [24] and the esterase from *Clostridium botulinum* Cbotu_EstA [25], which, to date, have not been assigned to any type, suggesting that Sis may represent a new type of PET hydrolase (Figure 2).

As shown in Figure 1b, the Sis model overlays with *Is*PETase (PDB 6QGC) [26], with an overall RMSD of 0.616 Å. The typical serine-hydrolase Ser-Asp-His catalytic triad conservation is visible in Figure 1c,d by the superimposition with *Is*PETase structure. In addition, the conservation of the position for the residues of the oxyanion hole, aromatic clamp, and disulfide bond is visible in Figure S5.

**Figure 3:**
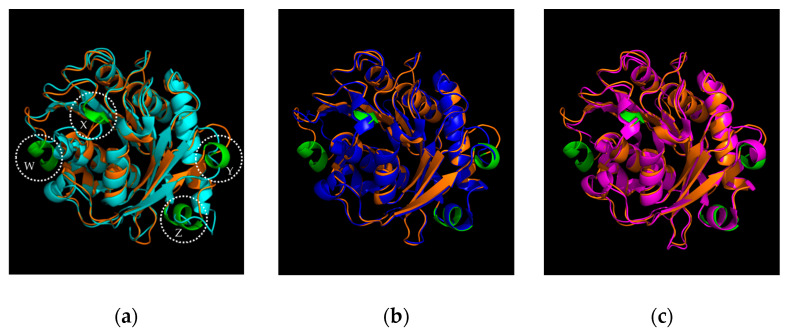
Structural superposition of Sis and (**a**) *Is*PETase (PDB 6QGC) with a highlight on the four α-helices, *W*, *X*, *Y*, and *Z*; (**b**) Thc_Cut1 (PDB 5LUI); (**c**) LCC (PDB 4EB0). In orange: Sis. In cyan: *Is*PETase. In blue: Thc_Cut1. In magenta: LCC. In green: the four α-helix structures.

Even though Sis shares a 46% identity with *Is*PETase, notable structural distinctions lie in the increased prevalence of α-helix formations in Sis, particularly evident in four specific regions (Ala262-Tyr267, Thr229-Asn231, Ser50-Cys53, and Ser133-Arg138) designated as regions W, X, Y, and Z, respectively, in Figure 3a. Three of the four α-helices mentioned, namely X, Y, and Z, were also found in Sis’ PAZy reference, the thermophilic Thc_Cut1 (PDB 5LUI) with the 52% of identity and with an overall RMSD of 0.436 Å (Figure 3b) [21]. 

The presence of these structures was also highlighted by the superimposition of Sis with another thermophilic PETase, the LCC (PDB 4EB0), whereby Sis displays an overall 44% identity and an overall RMSD of 0.621 Å [5] (Figure 3c). All the aminoacidic alignments are available in the Supplementary Material (Figures S1, S2, S4 and S6).

### 2.2. Determination of Optimal Reaction Conditions on pNP-Butyrate

Based on the DNA and protein alignments of Sis vs. *Is*PETase used as a comparison, as well as on the SignalP prediction (Figures S3, S4 and S7), the signal peptide of Sis [Met1-Val24] was excised (Figure 1a), and the putative PHE coding gene was then synthesised and inserted into the pET21b(+) vector, enabling the generation of a His-tagged protein. Consequently, the 267 amino acid sequence of Sis yielded a theoretical molecular weight of 28.4 kDa (Figure S8). The initial biochemical characterisation of recombinant Sis was performed on *para*-nitro-phenyl (*p*NP) butyrate, a substrate that mimics the ester bond of PET, as previously reported [11,19]. 

**Figure 4:**
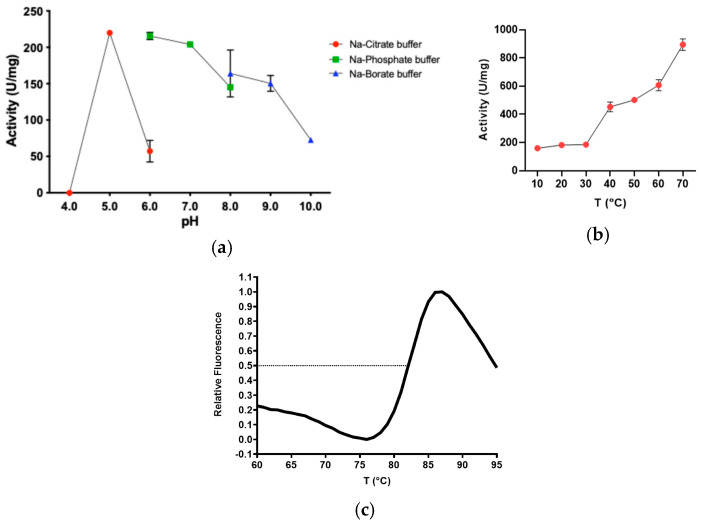
(**a**) Specific activity in terms of U/mg of Sis on *p*NP-butyrate depending on pH. All the assays were performed in quadruplicate, and the error bars indicate the standard error (*n* = 4). In red, buffer sodium-citrate; in green, buffer sodium-phosphate; in blue, buffer sodium-borate (**b**) Specific activity in terms of U/mg of Sis on *p*NP-butyrate depending on T. All the assays were performed in triplicate, and the error bars indicate the standard error (*n* = 3). (**c**) DSF analysis of Sis revealed a T_m_ of 82 °C.

Sis revealed a broad range of activity on *p*NP-butyrate. More in detail, the pH_opt_ of the reaction was observed in the range 5.0–7.0 (Figure 4a), and the T_opt_ was 70 °C (Figure 4b). Since a rapid spontaneous degradation of *p*NP-butyrate with the rising temperature was observed, the study of the effect of temperature on the enzymatic activity was performed up to 70 °C (Figure 4b). It is noteworthy that the difference in specific activity values measured at pH 6.0 using two different buffers is significant, with the activity in sodium-phosphate being 3.8-fold higher than in sodium-citrate. The structural stability of Sis was determined by using Differential Scanning Fluorimetry (DSF) analysis, revealing a T_m_ of 82 °C (Figure 4c) well above the optimal temperature. Sis was kinetically characterised on *p*NP-butyrate under optimal conditions of pH and temperature, revealing that the enzyme efficiently hydrolyses the substrate, with a k_cat_ of 447 ± 24 s^−1^, a K_M_ of 0.08 ± 0.01 mM, and a k_cat_/K_M_ ratio of 5587 s^−1^·mM^−1^. Considering the same substrate and the appropriate optimal reaction conditions, Sis exhibits a k_cat_/K_M_ 1.3-fold greater than *Is*PETase (4298 s^−1^·mM^−1^) [27]. Moreover, in comparison to the k_cat_/K_M_ ratios of LCC and Thc_Cut1, both obtained under the most favourable conditions (1630 s^−1^·mM^−1^ and 425 s^−1^·mM^−1^), Sis exhibits a specificity constant that is 3.4-fold and 13.1-fold higher, respectively [28,29].

**Figure 5:**
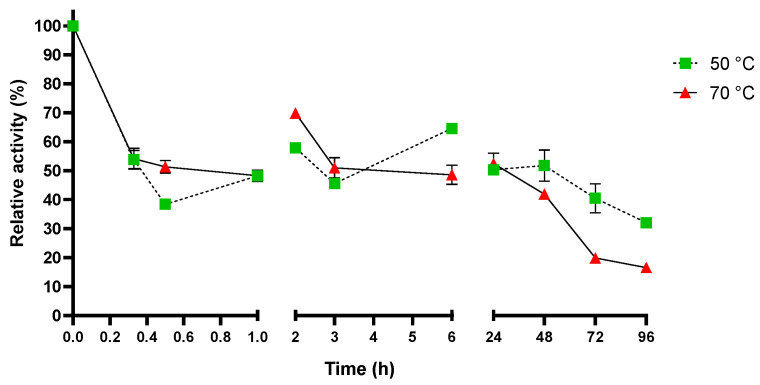
Functional thermostability of Sis after incubation at 50 °C and 70 °C. At each time period, specific activity on *p*NP-butyrate is measured under optimal reaction conditions. All the assays were performed in triplicate, and the error bars indicate the standard error (*n* = 3).

To assess the functional thermostability, the activity was evaluated under the optimal reaction conditions found (sodium-phosphate pH 7.0, 70 °C) on *p*NP-butyrate. Sis was incubated at two temperatures (50 °C and 70 °C) for up to 96 h (Figure 5). The enzyme displayed an overall good thermostability in both the conditions tested. More in detail, it kept around 60% of its activity after 30 min and approximately 50% after 24 h when incubated at both temperatures.

**Figure 6:**
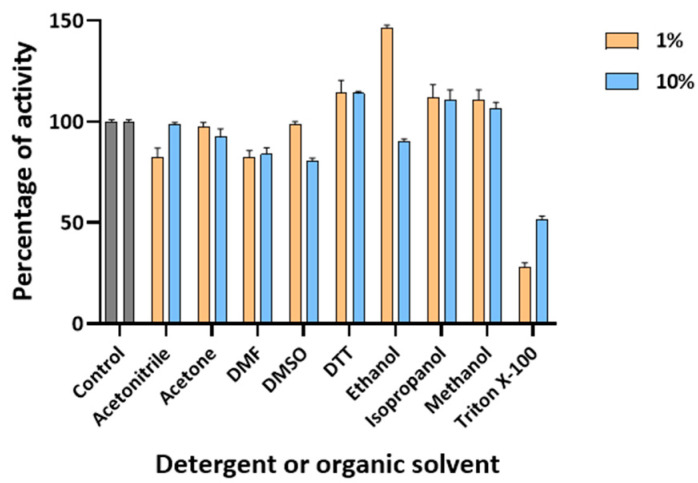
Effect of different detergents and organic solvents on Sis’ activity. All the assays were performed in quadruplicate, and the error bars indicate the standard error (*n* = 4) at standard conditions.

To assess the impact of detergents and organic solvents on Sis, its activity was also tested in the presence of varying concentrations (1% and 10%) of these additives. Sis displayed considerable tolerance to most of the additives examined. Specifically, 1% ethanol led to a 50% increase in enzymatic activity. In contrast, Triton X-100 reduced enzymatic activity by over 50% at both concentrations tested (Figure 6). It is interesting to note that this specific effect is different from what was previously reported for the *Is*PETase S92P/D157A mutant (9.9-fold more active than the wild type), which showed a reactivation of 9.3% in the presence of Triton X-100 and even 24.7% for *Is*MHETase [30].

### 2.3. Sis Can Degrade BHET and NanoPET at High Temperature

Preliminary tests were conducted using BHET and nanoPET (diameter < 0.22 μm) as substrates at 30 °C and 50 °C, employing a Phenol Red (PSP)-based assay. Sis exhibited significant enzymatic activity under both tested conditions. In addition, comparative assays with *Is*PETase confirmed higher activity for Sis at 50 °C, particularly noteworthy on nanoPET (Figure S9). In particular, Sis’ activity, measured as a ΔOD_558nm_/min signal, was 2.1-fold higher than the one measured for *Is*PETase (−0.0122 ΔOD_558nm_/min and −0.0058 OD_558nm_/min, respectively).

**Figure 7:**
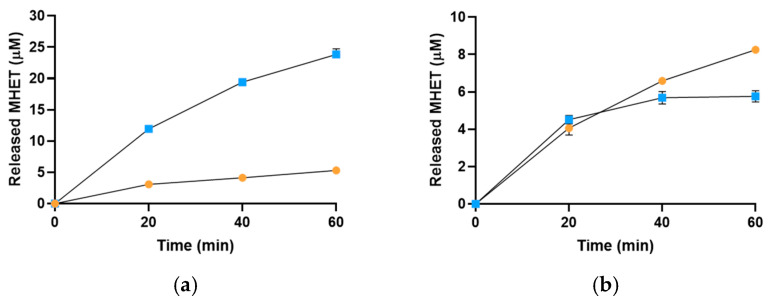

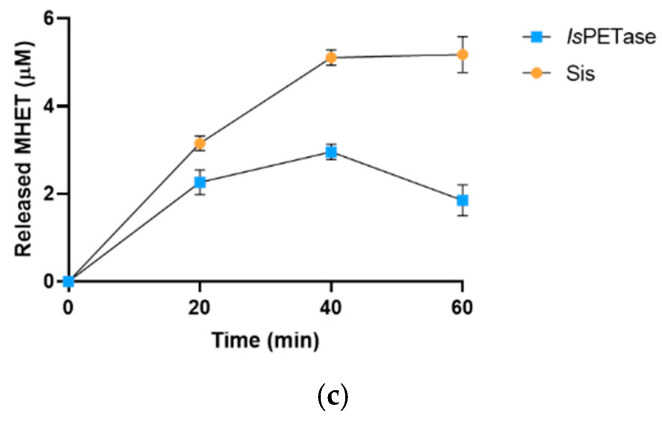
HPLC analysis of released MHET from the reaction on BHET by *Is*PETase or Sis at (**a**) 30 °C, (**b**) 50 °C, and (**c**) 70 °C. All the reactions were performed in triplicate, and the error bars indicate the standard error (*n* = 3).

The activity of Sis on BHET substrate was evaluated through reactions conducted at pH 7.0 and temperatures of 30 °C, 50 °C, and 70 °C, with *Is*PETase (pH_opt_ 6.5–8.0 and T_opt_ 30 °C for BHET degradation [31] and a T_m_ of ~49 °C [32]) used as a reference (Figure 7). While the analysis revealed a five-fold higher MHET release by *Is*PETase compared to Sis at 30 °C, Sis exhibited greater MHET production over *Is*PETase at 50 °C and 70 °C. More in detail, after 1 h of incubation at 50 °C, Sis produced 8.25 ± 0.05 μM MHET while *Is*PETase produced 5.76 ± 0.3 μM MHET, resulting in a 1.4-fold higher conversion for Sis vs. *Is*PETase in the same reaction conditions. Moreover, after 1 h of incubation at 70 °C, Sis produced 5.18 ± 0.41 μM MHET while *Is*PETase produced 1.85 ± 0.35 μM MHET, resulting in a 2.8-fold higher activity of Sis over *Is*PETase at this temperature (Figure 7). However, on BHET, *Is*PETase at 30 °C produced a higher concentration of MHET compared to Sis at its optimal conditions. This result may be attributed to a preference for BHET as substrate amplified to spontaneous degradation of MHET, which could reduce its chromatographic peak at higher temperatures.

**Figure 8:**
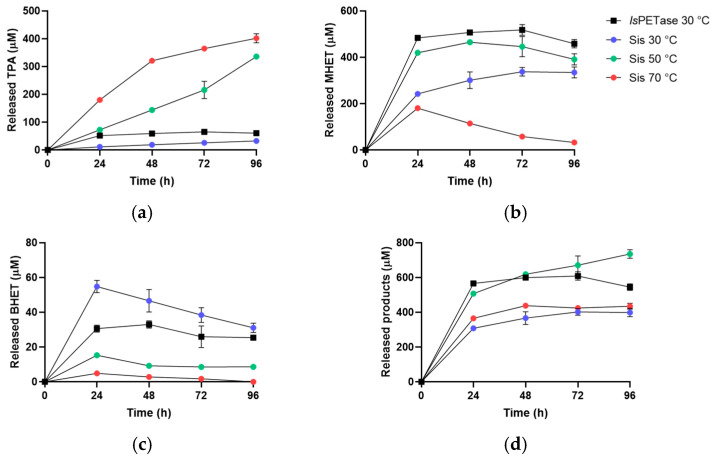
HPLC analysis of released (**a**) TPA, (**b**) MHET, (**c**) BHET, and (**d**) total released products from the reaction on nanoPET by *Is*PETase at 30 °C and by Sis at 30 °C, 50 °C, and 70 °C. All the reactions were performed in triplicate, and the error bars indicate the standard error (*n* = 3).

Finally, the activity of Sis on nanoPET was evaluated by HPLC monitoring the hydrolysis products (Figure 8 and Figure S10). After 24 h, the analysis of TPA concentration showed that Sis generated 3.5- and 1.5-fold more product at 70 °C and 50 °C, respectively, as compared to *Is*PETase under its optimal conditions. This disparity further widens after 96 h, reaching a 6.5-fold increase at 70 °C and a 5.5-fold increase at 50 °C (Figure 8a). After 48 h, a decrease in BHET and MHET concentrations was observed with the increasing temperature across all tested conditions, particularly pronounced for MHET at 70 °C, possibly because of the reduced stability of the product at higher temperatures (Figure 8b). The decrease in BHET concentration can be attributed to both spontaneous and enzymatic degradation of this compound, given that BHET is also a substrate of the two PETases (Figure 8c) [30]. The notable increase in total released products catalysed by Sis after 96 h at 50 °C, compared to that at 70 °C (Figure 8d), is possibly due to the production of TPA and MHET that cannot be solely explained by the spontaneous degradation of MHET and BHET (Figure 8a,b, respectively). The absence of a similar rise in total released products at 30 °C and 70 °C may be attributed to Sis’ thermophilicity and thermal stability (Figure 4b and Figure 5). These findings confirm the PETase activity of Sis and its thermophilic nature, illustrating its capability to hydrolyse nanoPET and to retain activity at 50 °C for up to four days.

## 3. Materials and Methods

### 3.1. Identification, Sequence Analysis, and Structural Prediction of New Putative PET Hydrolysing Enzymes

The in silico identification of novel putative PHE sequences was performed, as previously described [7,19]. Colabfold tool was used to predict the 3D structure [20], and SignalP 5.0 was exploited to predict the signal peptide and the relative cleavage site [33]. PyMOL v. 2.4.1 was used to detect the structural features and to visualise the superposition of 3D models. The Sis sequence is available in GenBank under the accession number PP704683.

### 3.2. Phylogenetic Analysis

The phylogenetic analysis was performed by using MEGA11 [34] with the Maximum Likelihood method and Whelan and Goldman model [35] and a discrete Gamma distribution to model evolutionary rate differences among sites (5 categories (+G, parameter = 2.3585)). The bootstrap consensus tree was produced by applying 1000 replicates [36]. The sequences used and their relative uniprot accession numbers are LCC (G9BY57), BhrPETase (A0A2H5Z9R5), Cbotu_EstA (A5I055), BsEstB (D7R6G8), *Is*PETase (A0A0K8P6T7), PET5 (R4YKL9), PET6 (A0A1Z2SIQ1), PET12 (A0A0G3BI90), RgPETase(A0A1W6L588), PaPETase (A0A1H6AD45), Lip1 (P19833), PET27 (A0A330MQ60), PET30 (A0A0C1F4U8), PET2 (C3RYL0), and PHL-7 (A0A165B1I1).

### 3.3. Construction of Expression Vectors

The gene coding for the new putative PHE Sis, lacking the signal peptide coding region, was codon-optimised for the expression in *E. coli*, exploiting the GenSmart™ Codon Optimization online tool (https://www.genscript.com/gensmart-free-gene-codon-optimization.html on 14 February 2023)) and synthesised in pET21b(+) plasmid by GenScript (GenScript Biotech Corporation, Piscataway, NJ, USA), giving a C-ter His-tagged protein. The gene coding for *Is*PETase, containing the nucleotide sequence corresponding to the signal peptide, was instead obtained by AddGene (Watertown, MA, USA) in pET21b(+) plasmid, giving a C-ter His-tagged protein. 

### 3.4. Heterologous Expression and Purification

The gene constructs were used to transform the *E. coli* BL21(DE3)-Codon Plus RIL strain. Cells were cultured at 37 °C in Luria-Bertani (LB) medium supplemented with 100 ng/μL ampicillin and 30 ng/μL chloramphenicol. When A_600 nm_ = 0.6 was reached, the protein expression was induced by adding 1 mM IPTG and incubating the flasks for 2 h at 37 °C for Sis and overnight at 18 °C for *Is*PETase [11]. Cells were withdrawn by centrifugation (5000× *g*, 20 min, 4 °C) and resuspended in lysis buffer (150 mM NaCl, 20 mM sodium-phosphate pH 7.3, 1% Triton X-100) in a ratio *w:v* = 1:4. Then, 25 mg/L lysozyme and 5 mg/L DNase I were added, followed by a freeze/thaw lysis. The Free Cell Extracts were clarified by centrifugation (17,000× *g*, 20 min, 4 °C) and filtered through a 0.45 µm PVDF membrane. The lysates were applied to a 1 mL HisTrap HP column exploiting an ÄKTApure chromatography system (Cytiva Life Sciences, Marlborough, MA, USA). The protein elution was performed by exploiting a discontinuous gradient of an imidazole-containing buffer (150 mM NaCl, 50 mM sodium-phosphate pH 7.3, 500 mM imidazole). The fractions containing the enzyme of interest were pooled, followed by dialysis (cutoff 12 kDa) overnight against 150 mM NaCl, 20 mM sodium-phosphate, and pH 7.3. The concentrations of the purified proteins were determined by Bradford assay, using Bio-Rad Protein Assay Dye Reagent Concentrate (Bio-Rad, Hercules, CA, USA) in transparent poly (methyl methacrylate) cuvettes (code 67.740 by Sarstedt, Nümbrecht, Germany) to a final volume of 1 mL for the mixture. 

### 3.5. Differential Scanning Fluorimetry

Sis (0.3 mg·mL^−1^) was mixed with 0.2 M sodium-phosphate pH 8.5 and 5X Sypro^TM^ Orange Protein Gel Stain by Invitrogen (Thermo Fisher Scientific, Waltham, MA, USA) to a final volume of 50 μL. The mix was prepared in triplicate and loaded into a clear white, sealed PCR 96-well plate in the StepOnePlus Real-Time PCR System by Applied Biosystems (Thermo Fisher Scientific, MA, USA). Samples were incubated at 20 °C for 10 min, then a melting scan from 20 °C to 95 °C with steps of 1 °C min^−1^ was recorded, with a final incubation at 95 °C for 10 min, as previously reported [19]. Protein unfolding was monitored by detecting changes in SYPRO™ Orange fluorescence. Fluorescence was normalised to the maximum value within each scan to obtain relative fluorescence. Melting temperatures were calculated as previously reported [37].

### 3.6. Biochemical Characterisation on Pnp-Vutyrate

Reactions containing Sis (0.05 μM/1.4 μg·mL^−1^) were incubated at 30 °C with 1 mM *p*NP-butyrate (dissolved in isopropanol) in 100 mM buffer sodium-citrate (pH 4.0–6.0), sodium-phosphate (pH 6.0–8.0), or sodium-borate (pH 8.0–10.0) in a final volume of 100 μL by using transparent polystyrene 96-well plates (code 82.1581.001 by Sarstedt, Nümbrecht, Germany). The release of *p*NP-OH was monitored continuously at 405 nm in quadruplicate, exploiting a BioTek Synergy HTX plate reader (Agilent Technologies, Santa Clara, CA, USA). The absorbance of *p*-nitrophenol at 30 °C and various pH values were measured using calibrated standard curves under reaction conditions.

To assess the thermophilicity, Sis (0.01 μM/0.28 μg·mL^−1^) was incubated with 1 mM *p*NP-butyrate in 100 mM sodium-phosphate buffer pH 7.0 at different temperatures, ranging from 10 °C to 70 °C. The absorbance change was recorded in triplicate in a final volume of 1 mL with a Cary 3500 Multicell UV-Vis Spectrophotometer equipped with a Peltier temperature controller (Agilent Technologies, CA, USA). The mM extinction coefficient of p-nitrophenol at a concentration of 7.0 mM and pH 7.0 was determined across the range of 10–70 °C: 10 °C (11.33 mM^−1^·cm^−1^), 20 °C (11.40 mM^−1^·cm^−1^), 30 °C (11.93 mM^−1^·cm^−1^), 40 °C (12.61 mM^−1^·cm^−1^), 50 °C (12.83 mM^−1^·cm^−1^), 60 °C (13.57 mM^−1^·cm^−1^), and 70 °C (14.05 mM^−1^·cm^−1^).

The effects of different detergents and organic solvents were evaluated by testing Sis (0.05 μM/1.4 μg·mL^−1^) at pH 7.0 and at 30 °C on 1 mM *p*NP-butyrate. For each detergent/solvent, final concentrations of 1% and 10% were tested. Each assay was performed in quadruplicate, using a reference blank without enzyme and a control without detergent/solvent. The release of *p*NP-OH was monitored continuously at 405 nm by using a plate reader, as reported above.

To evaluate the thermostability, Sis (0.05 μM/1.4 μg·mL^−1^) was incubated in 100 mM NaH_2_PO_4_ at pH 7.0 and at 50 °C and 70 °C for up to 4 days. The residual activity was measured in triplicate after 20 min, 30 min, 1 h, 2 h, 3 h, 6 h, and then every 24 h up to 96 h on 1 mM *p*NP-butyrate under the optimal reaction conditions (pH 7.0, 70 °C). The release of *p*NP-OH was spectrophotometrically measured, as reported above.

### 3.7. NanoPET Production

NanoPET was prepared as previously described [38,39]. Briefly, 0.25 g PET microplastics (maximum particle size 300 μm, crystallinity 40%, natural colour) by Goodfellow (Bad Nauheim, Germany) was dissolved in 25 mL 1,1,1,3,3,3-hexafluoro-2-propanol, in a ratio *w:v* = 1:100. The solution obtained was mixed under stirring and put drop by drop in 250 mL ddH_2_O. The solvent was removed by evaporation, heating the mixture up to 58.2 °C during the stirring. The bigger particles were removed by filtration (cutoff 0.22 μm), and the concentration was determined by weighing the nanoPET pellet obtained after the water evaporation exploiting Concentrator 5301 (Eppendorf, Hamburg, Germany).

### 3.8. Enzymatic Degradation of NanoPET through PSP Assay

Reactions on nanoPET were analysed by using the colorimetric assay based on PSP dye [38,39,40]. Briefly, the activity was spectrophotometrically detected continuously by A_558 nm_ decrease every 60 sec for 30 min at 30 °C and 50 °C in transparent 96-well plates (code 82.1581.001 by Sarstedt, Nümbrecht, Germany) by using a BioTek Synergy HTX plate reader (Agilent Technologies, CA, USA). Each enzyme (0.5 μM/1.4 μg·mL^−1^) was added to 0.2 mM PSP and 1 mM BHET (TCI, Tokyo Chemical Industry, Tokyo, Japan) or 0.2 mg·mL^−1^ nanoPET, up to a final volume of 200 μL by adding 1 mM sodium-phosphate buffer pH 8.0. All the assays were performed in duplicate, compared to an enzyme-free blank.

### 3.9. HPLC Analysis of Enzymatic Degradation of BHET and NanoPET

Sis and *Is*PETase were assayed by using 0.05 μM or 1.4 μg·mL^−1^ of each enzyme, respectively, on 150 μM BHET up to 1 h, and on 50 μg/mL nanoPET by using 0.01 μM or 0.28 μg·mL^−1^ of each enzyme, respectively, up to 5 days, in 0.1 M sodium-phosphate buffer pH 7.0. The experiment was set through empirical trials to obtain the clearest signal to be detected and to proceed with a clear conversion exploiting the pre-set time intervals. The activity on BHET was measured at 30 °C, 50 °C, and 70 °C for both the enzymes. The activity on nanoPET was instead assessed at 30 °C for *Is*PETase and at 30 °C, 50 °C, and 70 °C for Sis. For the time-course analysis, 50 μL of reaction was withdrawn and put on ice in a 150 μL final volume containing 15% acetonitrile and 0.1% trifluoroacetic acid (TFA). After centrifugation (15,000× *g*, 5 min, 4 °C), the supernatant was injected in a 20 μL loop and analysed by HPLC (LC-4000 Series System by Jasco, Oklahoma, OK, USA) equipped with a C18 column Reversed-phase, 4.6 mm × 250 mm, 5 μM particle size (Hamilton, NV, USA). The A_240 nm_ signal was monitored during the isocratic elution by using a solution acetonitrile:ddH_2_O (ratio 15:85), acidified with 0.1% TFA. BHET, MHET, and TPA were used as standards to evaluate the retention time and to quantify the products obtained. Every assay was performed in triplicate. 

## 4. Conclusions

Sis, a novel thermophilic PETase, exhibits several notable differences from other PET hydrolases, such as *Is*PETase, LCC, and Thc_Cut1. One of the most significant distinctions is its higher catalytic efficiency on general synthetic substrates as *p*NP-butyrate, demonstrated by a specificity constant, which surpasses that of *Is*PETase, LCC, and Thc_Cut1 by factors of 1.3, 3.4, and 13.1, respectively. Additionally, Sis’ high thermal stability, with a T_m_ of 82 °C, and its optimal activity at 70 °C make it particularly effective in degrading PET at temperatures close to PET’s glass transition temperature of ~75 °C. Indeed, at these temperatures, the polymer chains become more flexible and susceptible to hydrolysis, enabling Sis to convert PET directly to TPA and EG.

Moreover, the phylogenetic analysis indicates that Sis is phylogenetically distant from previously identified types of PHEs, suggesting it could possess a unique evolutionary story. This evolutive divergence likely contributes to its distinct features and properties, setting it apart from other known PHEs.

Sis’ ability to simplify the PET degradation process offers significant advantages for biotechnological applications. Its capability to function optimally at high temperatures is particularly beneficial for industrial-scale processes, where high reaction rates and enzyme stability are crucial. These attributes make Sis a promising candidate for enhancing the efficiency of PET recycling and upcycling processes and improving the management of PET waste on an industrial scale.

## Figures and Tables

**Table 1:** Thermostable PET hydrolases.

Name	Source	PET Hydrolase Type	T_m_ (°C)	Reference
BhrPETase	*bacterium HR29*	type I	101.0	[6]
LCC	metagenome	type I	86.2	[5]
PHL-7	metagenome	type I	79.1	[14]
PET2	metagenome	type IIa	69.0	[7]

## Data Availability

The original contributions presented in the study are included in the article/Supplementary Material, further inquiries can be directed to the corresponding author.

## Supplementary Materials

The supporting information can be downloaded at: https://www.mdpi.com/article/10.3390/ijms25158120/s1.
