# Supplementary material for: Biochemical Characterisation of Sis: A Distinct Thermophilic PETase with Enhanced NanoPET Substrate Hydrolysis and Thermal Stability
Source: Int J Mol Sci. 2024 Jul 25;25(15):8120. doi: 10.3390/ijms25158120 (PMC11311821; doi:10.3390/ijms25158120)
Supplement: Supplementary file 1 [file ijms-25-08120-s001.zip › ijms-3053989-supplementary.pdf]

Biochemical Characterisation of Sis: A Distinct Thermophilic PETase with Enhanced NanoPET Substrate Hydrolysis and Thermal Stability

Carmen Ercolano [1,†], Roberta Iacono [1,2,†], Valeria Cafaro [1], Elio Pizzo [1,3], Donato Giovannelli [1,2,4,5,6,7], Golo Feuerriegel [8], Wolfgang R. Streit [8], Andrea Strazzulli [1,2,*] and Marco Moracci [1,2]

1 Department of Biology, University of Naples Federico II, Complesso Universitario di Monte S. Angelo, Via Cinthia 21, 80126 Naples, Italy; carmen.ercolano@unina.it (C.E.); roberta.iacono@unina.it (R.I.); valeria.cafaro@unina.it (V.C.); elipizzo@unina.it (E.P.); donato.giovannelli@unina.it (D.G.); marco.moracci@unina.it (M.M.)
2 National Biodiversity Future Center (NBFC), 90133 Palermo, Italy
3 Centro Servizi Metrologici e Tecnologici Avanzati (CeSMA), University of Naples Federico II, 80126 Naples, Italy
4 Institute for Marine Biological Resources and Biotechnologies, Italian National Research Council, CNR-IRBIM, 60125 Ancona, Italy
5 Department of Marine and Coastal Science, Rutgers University, New Brunswick, NJ 08901, USA
6 Marine Chemistry and Geochemistry Department, Woods Hole Oceanographic Institution, Woods Hole, MA 02543, USA
7 Earth-Life Science Institute (ELSI), Tokyo Institute of Technology, Tokyo 152-8552, Japan
8 Department of Microbiology and Biotechnology, University of Hamburg, 22609 Hamburg, Germany; golo.feuerriegel@uni-hamburg.de (G.F.); wolfgang.streit@uni-hamburg.de (W.R.S.)
* Correspondence: andrea.strazzulli@unina.it
† These authors contributed equally to this work.

```
Sis            MKIVKTLLTVIFSVLMVVSMAHAVQIGPNPTSTLLN-GDGPFAVSSSSVSSLACGTGCAG     59
ADV92526.1     -------------------MANPYERGPNPTDALLEASSGPFSVSEENVSRL-SASGFGG     40
                                  **:  : *****.:**: ..***:**...** * ..:* .*

Sis            GTVYYPTTAGQYGVIAVCPGYTGTSSSIAWFARRLATHGFVTIAMDTNSTLDYPSSRATQ     119
ADV92526.1     GTIYYPRENNTYGAVAISPGYTGTEASIAWLGERIASHGFVVITIDTITTLDQPDSRAEQ     100
               **:***   . **.:*:.******.:****:..*:*:****.*::** :*** *.*** *

Sis            LAAALRYLINSSSSTIRSRIRTADRGVAGHSMGGGGTLIASADDSTLRVGIPMTPWNTYT     179
ADV92526.1     LNAALNHMINRASSTVRSRIDSSRLAVMGHSMGGGGTLRLASQRPDLKAAIPLTPWHLNK     160
               * ***.::** :***:**** ::  .* **********  :::   *:..**:***:  .

Sis            SFSSVRVPQMIFGADGDTIASASSHARPFYNSITQ-AEKAYALLNDASHFTPNSTDQRIG     238
ADV92526.1     NWSSVTVPTLIIGADLDTIAPVATHAKPFYNSLPSSISKAYLELDGATHFAPNIPNKIIG     220
               .:*** ** :*:*** **** .::**:*****: .  .***  *:.*:**:**  :: **

Sis            RYGVAFAKRFVDGDTRYNPFLCGAEHTAYATSSRFDTYLSNCPY     282
ADV92526.1     KYSVAWLKRFVDNDTRYTQFLCPGPRDGL--FGEVEEYRSTCPF     262
               :*.**: *****.****. *** . : .    ...: * *.**:
```

Figure S1. Aminoacidic alignment of Sis and PAZy reference.

```
Sis                                  MKIVKTLLTVIFSVLMVVSMAHAVQIGPNPTSTLLNGDGPFAVSSSSVSSLACGTGCAGG     60
MBP7342671.1_MDD4241650.1_NLX52001.1 MKVWKVLLASIFTLLLTVSLAHAVQIGPAPTSTLLNGDGPFAVSSSSVSSWVT--GFGGG     58
                                     **: *.**: **::*:.**:******** ********************  .   * .**

Sis                                  TVYYPTTAGQYGVIAVCPGYTGTSSSIAWFARRLATHGFVTIAMDTNSTLDYPSSRATQL     120
MBP7342671.1_MDD4241650.1_NLX52001.1 TIYYPTTSGQYAAVAVCPGFTGTSSSISWFARRLATHGFVTIAMNTNTIYDYPSSRATQL     118
                                     *:*****:***..:*****:*******:****************:**:  **********

Sis                                  AAALRYLINSSSSTIRSRIRTADRGVAGHSMGGGGTLIASADDSTLRVGIPMTPWNTYTS     180
MBP7342671.1_MDD4241650.1_NLX52001.1 AAALRYLLNSSSSTIRARIRTADRAVAGHSMGGGGTLIASANDSTLRAGIPLTPWNTYTS     178
                                     *******:********:*******.***************:*****.***:********

Sis                                  FSSVRVPQMIFGADGDTIASASSHARPFYNSITQAEKAYALLNDASHFTPNSTDQRIGRY     240
MBP7342671.1_MDD4241650.1_NLX52001.1 FSSVRVPQMIFGADGDTIAPYASHARPFYNSLSYPEKAYALLNGATHFTPNSTDQRIGRY     238
                                     *******************  :*********::  ********.*:**************

Sis                                  GVAFAKRFVDGDTRYNPFLCGAEHTAYATSSRFDTYLSNCPY   282
MBP7342671.1_MDD4241650.1_NLX52001.1 GVAFAKRFVDGDTRYTPFLCGAEHTAYATSLRFDTYLSTCPY   280
                                     ***************.************** *******.***
```

Figure S2. Aminoacidic alignment of Sis and *NR* references.

```
IsPETase        ATGAACTTCC---CCCGTGCCTCGCGCCTTATGCAGGCTGCTGTGCTGGGCGGCCTTATG  57
Sis             ATGAAAATTGTTAAAACTTTATTAACAGTAATTTTCAGTGTTTTGATGGTTGTCAGTATG  60
                *****  *         *   *      * **      ** * ** ***  * *  ****

IsPETase        GCCGTTTCCGCAGCGGCCACCGCGCAGACCAATCCGTATGCGCGGCCCCAACCCTACC  117
Sis             GCCCA------------CGCGGTGCAGATC---------------GGACCCAATCCGACA  93
                ***              * * * ***** *               ** ***** ** **

IsPETase        GCCGCCTCGTTGGAA----GCCAGCGCGGGACCCTTTACCGTTCGTAGCTTTAC--CGTT  171
Sis             TCCACCCTGCTGAATGGTGACGGGCCTTTCGCGGTATCCTCGTCCAGCGTCTCCAGTCTT  153
                 ** **  * ** *      *  **      *  * * *   **     * * *    **

IsPETase        AGCCGTCCGTCCGGATATGGTGCAGGGACCGTCTATTACCCAACCAATGCAGGCGGCACC  231
Sis             GCTTGCGGTACTGGTTGCGCCGGCGGCACGGTCTATTACCCAACTACGGC---CGGTCAG  210
                    *     * ** *  *  *  ** ** ************** *  **   ***

IsPETase        GTTGGCGCGATTGCAATCGTCCCCGGGTACACCGCGCGTCAAAGCAGCATTAAGTGGTGG  291
Sis             TATGGCGTAATCGCGGTTTGCCCGGGCTATACCGGAACAAGCTCTTCGATTGCCTGGTTT  270
                  *****  ** **  *   *** ** ** ****              ***   ****

IsPETase        GGTCCGCGCTTAGCTAGCCATGGCTTTGTGGTTATTACCATCGATACGAACAGCACTCTA  351
Sis             GCCCGCAGATTGGCTACGCATGGCTTTGTGACCATTGCCATGGACACAAACAGCACTTTG  330
                *  *   * ** ****  ************   *** **** ** ** ********* *

IsPETase        GACCAGCCCAGCAGCCGTAGCTCGCAACAGATGGCCGCGCTTCGTCAAGTTGCGAGCTTG  411
Sis             GATTATCCGTCGAGCCGCGCCACGCAGTTGGCGGCAGCGCTCAGATACCTGATCAATTCA  390
                **  * **    *****   * ****   *  *** *****  *  *  *    *  *

IsPETase        AACGGGACCAGCAGTAGCCCGATTTACGGAAAGGTCGATACTGCCCGCATGGGTGTGATG  471
Sis             TCGAGCAGCAC---------CATCCGTTCCCGCATCCGCACCGCTGACCGCGGCGTGGCG  441
                    * * **           **           **   ** **   *   ** ***  *

IsPETase        GGCTGGTCAATGGGGGGCGGCGGTTCACTTATTAGCGCCGCGAACAACCCGAGTTTAAAA  531
Sis             GGGCATTCCATGGGCGGCGGCGGAACTTTGATTGCTTCCGCGGATGATTCCACTTTAAGA  501
                **    ** ***** ********  *  * ***    ***** *  *  * * ***** *

IsPETase        GCAGCGGCACCGCAGGCGCCATGGGACTCTTCAACCAACTTCAGCAGTGTTACCGTGCCG  591
Sis             GTCGGCATTCCCATGACGCCGTGGAACACCTACACCAGCTTCTCCTCGGTGCGTGTGCCG  561
                *  *     **   * **** *** ** * *  **** ****  *   **    ******

IsPETase        ACGCTGATTTTCGCGTGCGAGAATGATAGCATTGCACCGGTGAACAGCAGCGCGCTGCCG  651
Sis             CAGATGATATTCGGCGCGGACGGCGACACCATTGCTTCCGCTTCATCCCACGCGCGTCCG  621
                  * **** ****     **    ** * ******  * *       *  *****  ***

IsPETase        ATTTATGATAGCATGTCCCGCAACGCAAAACAGTTTCTGGAAATTAACGGCGGTAGCCAC  711
Sis             TTCTACAACAGCATCACTCAGGCCGAAAAGGCGTATGCTCTGTTGAATGACGCGTCGCAT  681
                 * **  * *****  * *    ** ***   ** *       * ** * **     **

IsPETase        TCTTGTGCCAACTCTGGGAACAGCAACCAGGCACTGATCGGAAAAAAAGGGGTTGCATGG  771
Sis             ---------TTCACGCCAAACAGCACGGATCAACGCATCGGGCGCTATGGCGTGGCTTTT  732
                           * *    *******   *   **  *****     * ** ** ** *

IsPETase        ATGAAACGATTCATGGATAATGACACCCGTTACTCAACCTTCGCCTGTGAGAATCCCAAC  831
Sis             GCCAAACGTTTTGTGGACGGCGATACCCGCTACAATCCGTTCCTGTGCGGCGCTGAGCAT  792
                   ***** **  ****    ** ***** ***    * ***   ** *    *    *

IsPETase        AGCACACGCGTGTCGGATTTTCGCACCGCGAAC-----------TGTTCCTGA----    873
Sis             ACTGCTTACGCGACCAGTTCACGCTTTGATACCTACCTGTCAAATTGTCCTTACTAA    849
                *   *   ** * *   **  ***   *  * *           *  **** *
```

Figure S3. Nucleotide alignment of Sis and *Is*PETase.

```
IsPETase        MNFPRASRLMQAAVLGGLMAVSAAATAQTNPYARGPNPTAASLEASAGPFTVRSFTVS--    58
Sis             MK---IVKTLLTVIFSVLMVVSMA-----HAVQIGPNPTSTL-LNGDGPFAVSSSSVSSL    51
                *:     : : :.::. **.** *     :    *****::    . ***:* * :**

                                             ♣
                                             ♦
IsPETase        -RPSGYGAGTVYYPTNAGGTVGAIAIVPGYTARQSSIKWWGPRLASHGFVVITIDTNSTL    117
Sis             ACGTGCAGGTVYYPTTA-GQYGVIAVCPGYTGTSSSIAWFARRLATHGFVTIAMDTNSTL    110
                   :* ..*******.* *  *.**: ****. .*** *:. ***:****.*::******

                                                          ♥♣
IsPETase        DQPSSRSSQQMAALRQVASLNGTSSSPIYGKVDTARMGVMGWSMGGGGSLISAANNPSLK    177
Sis             DYPSSRATQLAAALRYLI---NSSSSTIRSRIRTADRGVAGHSMGGGGTLIASADDSTLR    167
                * ****::*  **** :    .:*** * .:: **  ** * ******:**::*:: :*:

                       ♦                    ♥ ♦                            ♥
IsPETase        AAAPQAPWDSSTNFSSVTVPTLIFACENDSIAPVNSSALPIYDSMSRNAKQFLEINGGSH    237
Sis             VGIPMTPWNTYTSFSSVRVPQMIFGADGDTIASASSHARPFYNSITQAEKAYALLNDASH    227
                .. * :**:: *.**** ** :**..:.*:** ..* * *:*:*:::  * :  :*..**

                                                   ♠                   ♠
IsPETase        SCANSGNSNQALIGKKGVAWMKRFMDNDTRYSTFACENPNSTRVSDF----RTANCS--     290
Sis             FTPN---STDQRIGRYGVAFAKRFVDGDTRYNPFLCGAEHTAYATSSRFDTYLSNCPY      282
                   *   *.:  **: ***: ***:*.****. * *   ::: .:.       :**
```

In cyan: signal peptide sequence of *Is*PETase

In green: GXSXG motif

♣: Oxyanion hole

♦: Aromatic clamp

♥: Catalytic triad

♠: Disulfide bond

Figure S4. Aminoacidic alignment of Sis and *Is*PETase, with a focus on the conserved motifs.

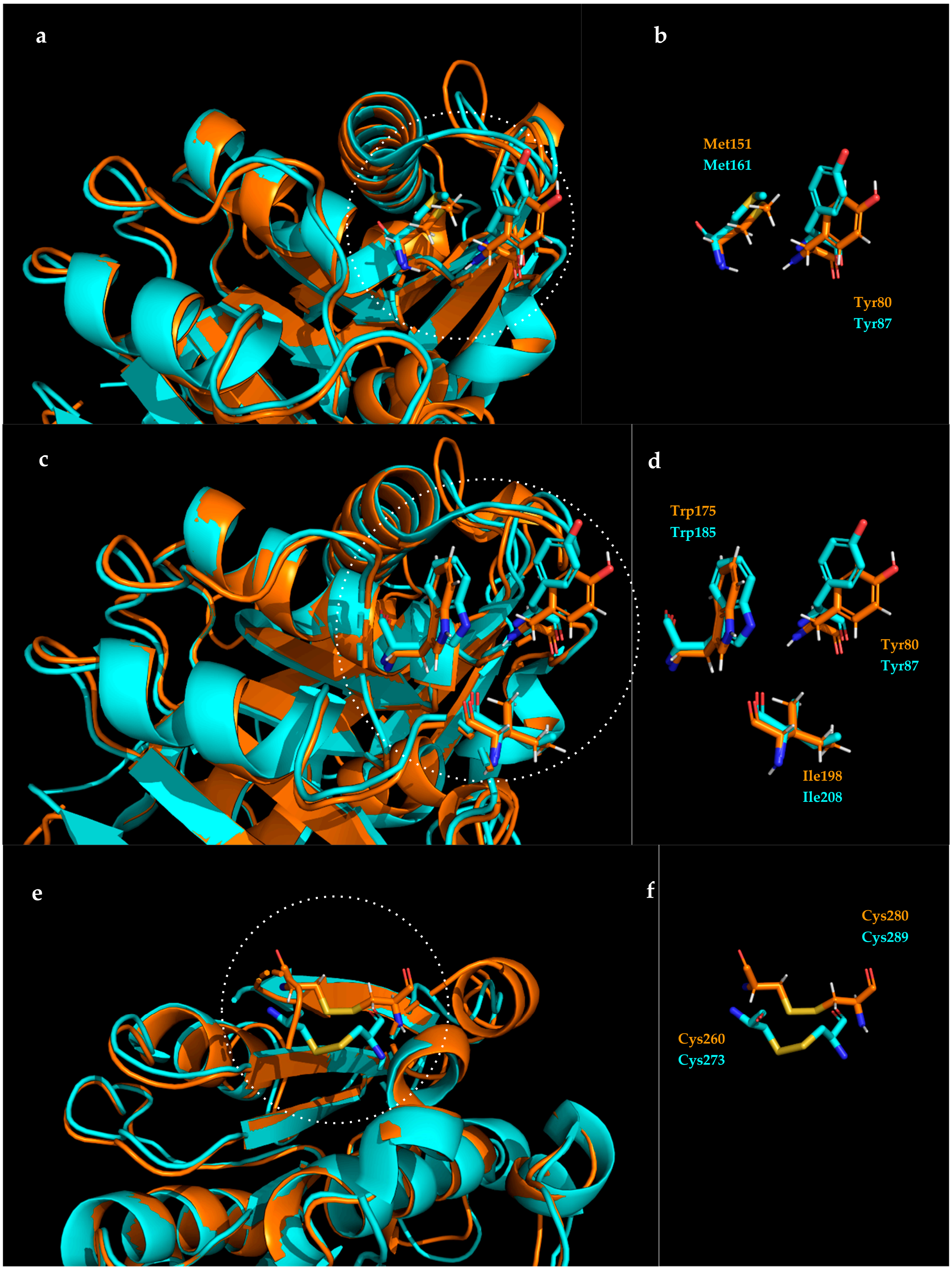

Figure S5. Structural superimposition of Sis (orange) and *Is*PETase (cyan, PDB 6QGC), with a focus on residues of (**a-b**) oxyanion hole, (**c-d**) aromatic clamp, and (**e-f**) disulfide bond. Residues are highlighted in cyan for *Is*PETase and in orange for Sis, and non-carbon atoms are coloured according to the CPK colouring convention.

```
Sis             ----------MKIVKT-----LLTVIFSVLMVVSMAHAVQIGPNPTSTLLNGDGPFAVSS       45
AEV21261.1      MDGVLWRVRTAALMAALLALAAWALVWASPSVEAQSNPYQRGPNPTRSALTADGPFSVAT       60
                            :: :       :::::   * : ::  * ***** : *..****:*::

Sis             SSVSSLACGTGCAGGTVYYPTTA-GQYGVIAVCPGYTGTSSSIAWFARRLATHGFVTIAM       104
AEV21261.1      YTVSRLSV-SGFGGGVIYYPTGTSLTFGGIAMSPGYTADASSLAWLGRRLASHGFVVLVI       119
                 :** *:  :* .**.:**** :   :* **:.****. :**:**:.****:****.:.:

Sis             DTNSTLDYPSSRATQLAAALRYLINSSSSTIRSRIRTADRGVAGHSMGGGGTLIASADDS       164
AEV21261.1      NTNSRFDYPDSRASQLSAALNYLRTSSPSAVRARLDANRLAVAGHSMGGGGTLRIAEQNP       179
                :*** :***.***:**:***.** .** *::*:*: :   .************  : ::

Sis             TLRVGIPMTPWNTYTSFSSVRVPQMIFGADGDTIASASSHARPFYNSITQ-AEKAYALLN       223
AEV21261.1      SLKAAVPLTPWHTDKTFN-TSVPVLIVGAEADTVAPVSQHAIPFYQNLPSTTPKVYVELD       238
                :*:..:*:***:* .:*. . ** :*.**:.**:* .*.** ***:.: . : *.*. *:

Sis             DASHFTPNSTDQRIGRYGVAFAKRFVDGDTRYNPFLCGAEHTAYATSSRFDTYLSNCPY        282
AEV21261.1      NASHFAPNSNNAAISVYTISWMKLWVDNDTRYRQFLCNVNDPALS---DFRTNNRHCQ-        293
                :****:***.:  *. * ::: * :**.****. ***..:. * :    * *   :*
```

Figure S6. Aminoacidic alignment of Sis and LCC.

Sequence

Prediction: Signal peptide (Sec/SPI)

Cleavage site between pos. 23 and 24: AHA-VQ. Probability: 0.9802

**Protein type**	**Signal peptide (Sec/SPI)**	**TAT signal peptide (Tat/SPI)**	**Lipoprotein signal peptide (Sec/SPII)**	**Other**
**Likelihood**	0.998	0.0004	0.0009	0.0007

Download: PNG / EPS / Tabular

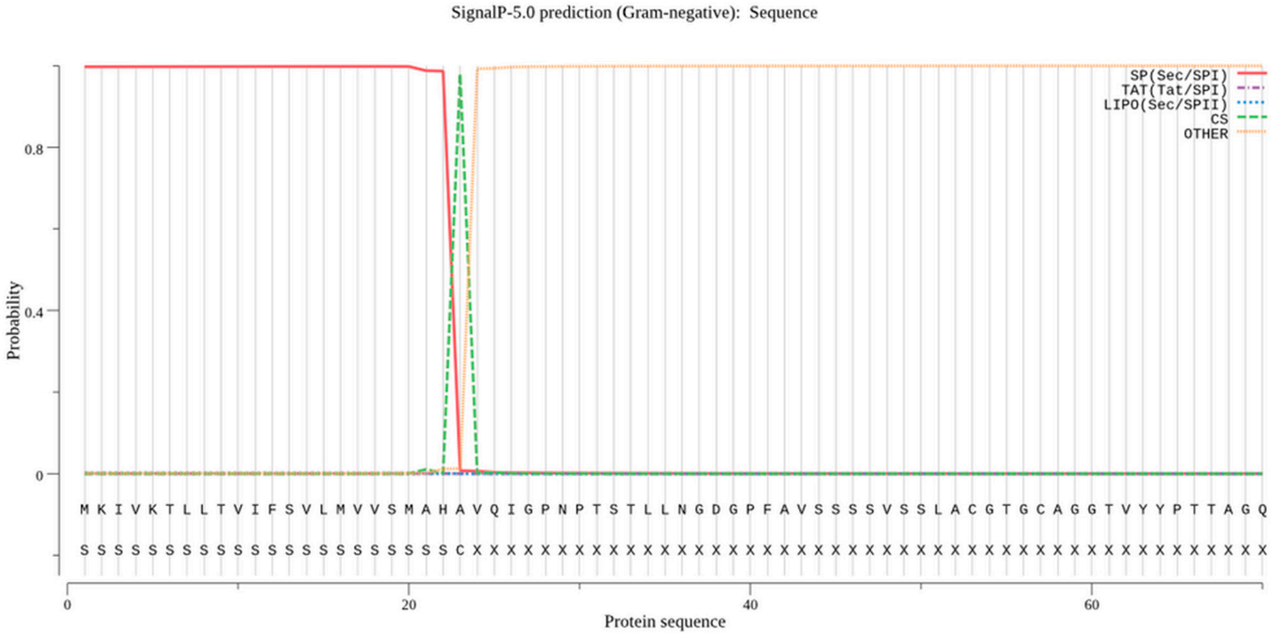

Figure S7. Signal peptide prediction using SignalP, indicating a cleavage between aa positions 23 and 24.

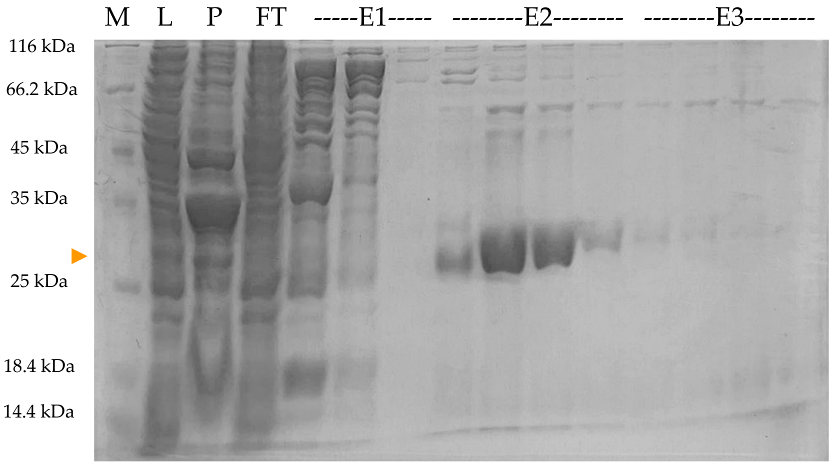

Figure S8. SDS-PAGE (10%) concerning the purification of Sis produced in *E. coli* BL21(DE3)-Codon Plus RIL cells. M = Marker Thermo Scientific™ Pierce™ Unstained Protein MW Marker (Catalog number: 26610); L = cell lysate; P = pellet from lysis procedure (residual after centrifugation to obtain the cell lysate); FT = flow-through of Histrap purification; E1 = Histrap fractions of 20 mM imidazole elution; E2 = Histrap fractions of 250 mM imidazole elution; E3 = Histrap fractions of 500 mM imidazole elution. The molecular weight of Sis is 28.4 kDa: the expected migration height is indicated with an orange arrow.

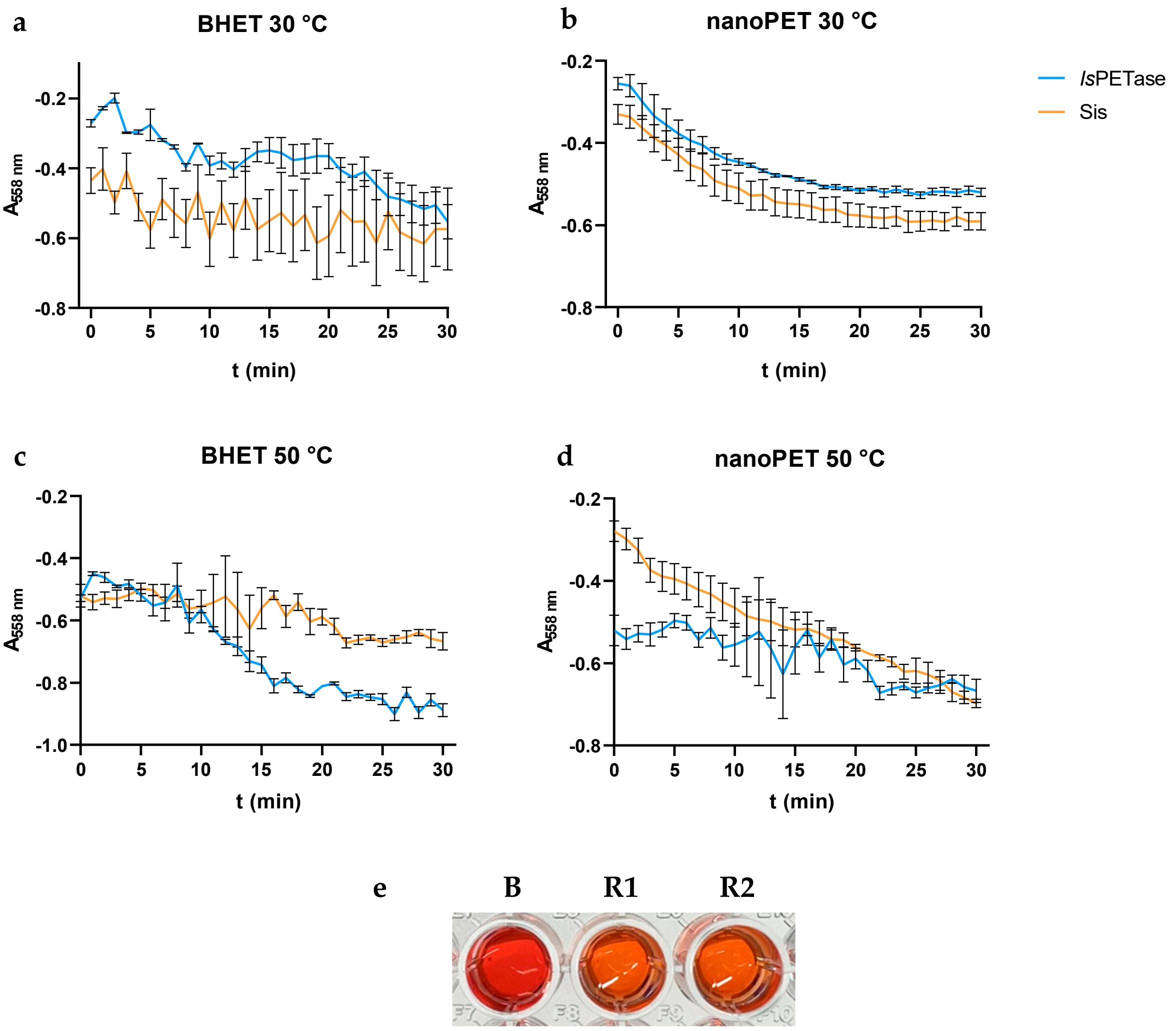

Figure S9. PSP assay of Sis and *Is*PETase on BHET and nanoPET. Decrease of $A_{558\ nm}$ signal measured every 60 sec for 30 min on (**a**) BHET at 30 °C, (**b**) nanoPET at 30 °C, (**c**) BHET at 50 °C and (**d**) nanoPET at 50 °C. (**e**) Example of visive change of reaction colour produced by Sis on nanoPET after 30 min of incubation at 50 °C with nanoPET, performed in duplicate (R1 and R2). A shift from red to orange is observed if comparing B (Blank with no enzyme), R1 and R2.

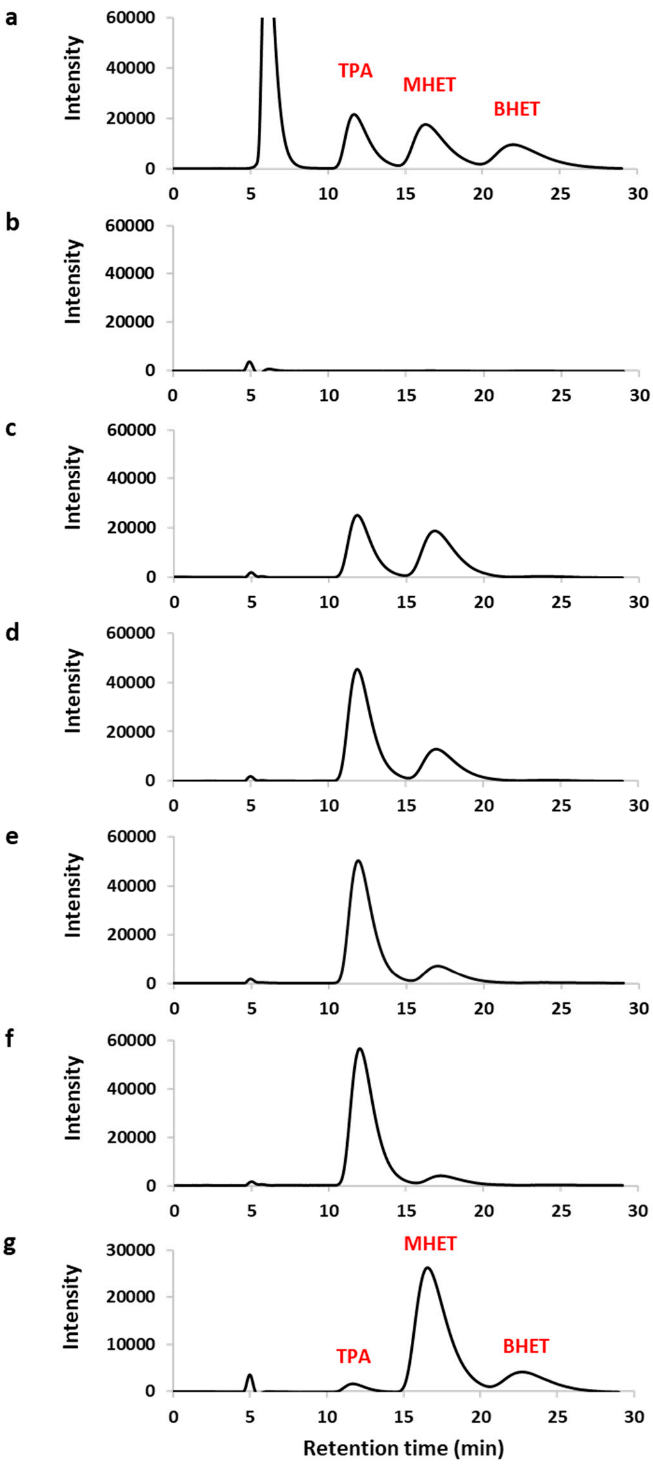

Figure S10. Time-course analysis of released products from the reaction of Sis on nanoPET by Reverse-Phase HPLC. Activity of Sis was monitored over the time at 70 °C and representative chromatograms were reported. Products were separated on a C18 column by isocratic elution (15% acetonitrile and 0.1% TFA) with a flow rate of 0.5 mL/min, recording the absorbance at 240 nm (separation length: 30 min). TPA, MHET and BHET were used as standards to evaluate the retention times (11.7, 16.3 and 21.9 min, respectively) of the hydrolysis products (a). Reaction at 70 °C was monitored at the start of incubation (b), after 24 h (c), 48 h (d), 72 h (e) and 96 h (f). Activity of Sis was also monitored at 30 °C: representative chromatogram at 24 h incubation time was reported to highlight all three reaction products (g).
